# Effective Combination Immunotherapy with Oncolytic Adenovirus and Anti-PD-1 for Treatment of Human and Murine Ovarian Cancers

**DOI:** 10.3390/diseases10030052

**Published:** 2022-08-08

**Authors:** Camilla Heiniö, James Clubb, Tatiana Kudling, Dafne Quixabeira, Victor Cervera-Carrascon, Riikka Havunen, Susanna Grönberg-Vähä-Koskela, João Manuel Santos, Johanna Tapper, Anna Kanerva, Akseli Hemminki

**Affiliations:** 1 Cancer Gene Therapy Group, Faculty of Medicine, TRIMM, RPU, University of Helsinki, 00270 Helsinki, Finland; 2 TILT Biotherapeutics Ltd., 00270 Helsinki, Finland; 3 Comprehensive Cancer Center, Helsinki University Hospital, 00270 Helsinki, Finland; 4 Department of Obstetrics and Gynecology, Helsinki University Central Hospital, 00270 Helsinki, Finland

**Keywords:** ovarian cancer, oncolytic virus, immune checkpoint inhibitor, adenovirus, TILT-123

## Abstract

**Simple Summary:**

This study was conducted to find a new, more efficient, treatment for ovarian cancer. A combination of an oncolytic adenovirus (TILT-123) with immune checkpoint inhibitors was employed to treat ex vivo patient samples and was found statistically significantly more effective than control treatments ex vivo and showed potent efficacy towards in vivo tumor growth.

**Abstract:**

Ovarian cancer (OvCa) is one of the most common gynecological cancers and has the highest mortality in this category. Tumors are often detected late, and unfortunately over 70% of OvCa patients experience relapse after first-line treatments. OvCa has shown low response rates to immune checkpoint inhibitor (ICI) treatments, thus leaving room for improvement. We have shown that oncolytic adenoviral therapy with Ad5/3-E2F-d24-hTNFa-IRES-hIL2 (aka. TILT-123) is promising for single-agent treatment of cancer, but also for sensitizing tumors for T-cell dependent immunotherapy approaches, such as ICI treatments. Therefore, this study set out to determine the effect of inhibition of the immune checkpoint inhibitors (ICI), in the context of TILT-123 therapy of OvCa. We show that simultaneous treatment of patient derived samples with TILT-123 and ICIs anti-PD-1 or anti-PD-L1 efficiently reduced overall viability. The combinations induced T cell activation, T cells expressed activation markers more often, and the treatment caused positive microenvironment changes, measured by flow cytometric assays. Furthermore, in an immunocompetent in vivo C57BL/6NHsda mouse model, tumor growth was hindered, when treated with TILT-123, ICI or both. Taken together, this study provides a rationale for using TILT-123 virotherapy in combination with TILT-123 and immune checkpoint inhibitors together in an ovarian cancer OvCa clinical trial.

## 1. Introduction

Every cancer type has unique characteristics that should be taken into consideration for successful treatment outcomes. For ovarian cancer (OvCa), challenges often lie in the frequently disseminated disease and suppressive tumor microenvironment [1]. Amongst other cells, adipocytes, tissue resident macrophages and myeloid derived suppressor cells (MDSCs) in the omentum and/or in the tumor secrete growth factors and other bioactive molecules that facilitate tumor cell and regulatory T cell (Treg) proliferation and viability and hinder T cell cytotoxicity functions [2,3]. Collectively, this creates a tumor environment that supports tumor growth and treatment resistance and makes the tumor difficult to cure.

Immune checkpoint inhibitors (ICIs) targeting programmed cell death-1 (PD-1), programmed cell death-ligand 1 (PD-L1) or cytotoxic T lymphocyte-associated antigen-4 (CTLA-4) have become widely studied and used immunotherapies because of their impressive clinical results in some cancer types, such as melanoma. For example, in a Phase III study of Stage III melanoma, the authors reported an improved 5-year recurrence-free survival (40.8% vs. 30.3% with placebo) with ipilimumab (anti-CTLA4), and a 5-year overall survival of 65.4% vs. 54.4% with placebo [4], thus leading to the FDA approval for ipilimumab for adjuvant therapy of melanoma.

In another phase II study, investigators enrolled patients with relapsed/refractory classic Hodgkins lymphoma after autologous hematopoietic cell transplantation treatment failure (in regard to brentuximab vedotin treatments). All patients received nivolumab (anti-PD-1) 3 mg/kg every 2 weeks until disease progression/unacceptable toxicity. After a median follow-up of 1.5 years, the objective response rate varied between 65% to 73% between treated groups [5].

However, the clinical efficacy of ICI is not as impressive in all cancer types and thus biomarkers indicating efficient use of ICI have been a relevant field of study [6]. In ovarian cancer the efficacy of ICI is hampered due to the aforementioned suppressive environment and often late diagnosis [7].

Taking this into consideration, it is evident that new therapies need to be developed. From an immunological point of view, it is promising that some tumor types such as epithelial ovarian cancers express many known tumor-associated and mutational antigens (Tumor associated antigens (TAAs) or neo-antigens, respectively), and some tumors are infiltrated by lymphocytes (TILs) [8,9,10]. However, this is not always the case, and in a comparison made by Alexandrov et al. it was reported that OvCa has fewer somatic mutations compared to tumor types that have high response rates to ICI (placed 15/30 among analysed cancer types) [11]. This is underlined by the fact that, despite the success of immunotherapy in other malignancies, ICI for epithelial ovarian serous cancer has only resulted in modest results so far, with median response rates usually varying from 10% up to 15% [12].

There is some evidence indicating that the lack of tumor-infiltrating cytotoxic lymphocytes and chemokines for T cell recruitment significantly reduces the antitumor effects of ICIs [13]. Therefore, oncolytic viruses (OVs) have emerged as a way to boost immunotherapy. They are promising tools as selective replication and direct oncolysis in tumor cells are coupled with the successful recruitment of immune cells [14]. Nevertheless, it has been speculated that single treatments have limited therapeutic efficacy due to intrinsic immune regulating counter reaction that limits the effect of recruited immune cells [15]. However, for example talimogene laherparepvec, an oncolytic herpes virus, has already received FDA and EMA approval for use in certain stages of melanoma [16], showing the positive effects of virotherapy. Clinical trials on oncolytic viruses as a single agent therapy for treatment of ovarian cancer have been started, not only with adenoviruses but with others too. For example reo-, vaccinia- and measles-viruses have entered clinical trials [17]. An oncolytic measles virus called MV-NIS, modified to express the sodium iodine symporter, had dose dependent effects in an clinical evaluation and was well tolerated [18]. Thus, to compensate for the modest results of single treatments, the combination of OVs and ICIs may be a reasonable and promising strategy to synergistically overcome immunosuppression in the TME [15]. Subsequently, in this study, an oncolytic adenovirus called TILT-123 that was created to be synergistically employed together with adoptive T cell therapy [14,19] was used in combination with ICI. This was studied in an ovarian cancer setting. Thus, the study rationale was that the cytokines coded by the virus, TNFa and IL2, would enhance T cell recruitment, proliferation and activation, while the ICI would plausibly retain an active antitumor immune reaction. Thus, the ICI combination would enable avoidance of the normally inevitable immune suppressive counteraction towards the virus and ICI. Furthermore, in support of our hypothesis, it has been shown that the combination of checkpoint inhibitors and TILT-123 work synergistically. More specifically, Cervera-Carrascon et al. showed enhanced efficacy and positive tumor microenvironment changes [20].

In this study we demonstrate that TILT-123 therapy with or without ICI caused a TME change by inducing pro-inflammatory cytokine release. The combinatory treatment with anti-PD-1 produced an activated T cell phenotype (in both CD4+ and CD8+ cells), characterized by higher granzyme B, Lamp-1 and CD69+ expression on T cells. Additionally, mice treated with TILT-123 and ICI anti-PD-1 showed tumor growth reduction in both subcutaneous and intraperitoneal tumors.

## 2. Materials and Methods

### 2.1. Cell Lines and Viruses

ID8-luc2 cells were obtained as a kind gift from Dr. Mary L. Disis, University of Washington. They were grown in standard conditions in supplemented media; Dulbecco’s Modified Eagle Medium(DMEM, Biowest, Riverside, MO, USA) + 4% FBS, 1% insulin-transferin-selenite, 1% L-glutamine (Sigma, Maryland Heights, MO, USA), 1% penicillin/streptomycin (Cytive, Marlborough, MA, USA) in a humidified incubator at 37 °C.

The TILT-123 virus was created using the Ad5/3-E2F-d24 as backbone. Transgenes were inserted using a BAC recombineering system described in [14]. The built adenovirus has a backbone of Ad5/3-E2F-d24 (OAd) carrying human IL-2 (hIL2) and hTNFa. Two modifications renders the virus replication tumor specific: an E2F promoter and a 24-base pair (bp) deletion in the constant region 2 of *E1A*.

The construction of replication-deficient adenoviruses Ad5-CMV-mIL-2 and Ad5-CMV-mTNF-α, was carried out as described previously [14,21]. In short, Ad5-CMV-mTNFα or mIL2 were constructed by inserting expression cassettes with murine cytokines into the multiple cloning site of the shuttle plasmid pDC315 (AdMax, Microbix Biosystems, Mississauga, ON, Canada). Shuttle plasmids were recombined with pBHGloxdelE13cre (AdMax), which carries the whole adenovirus genome, and resulting rescue plasmids were transfected to 293 cells to generate the final virus constructs.

### 2.2. Patient Material and Processing

Patient samples that were used for this study are listed in Table 1. The samples were collected from OvCa patients at the department of obstetrics and gynecology, Helsinki University Central Hospital in 2021. The samples were collected from patients undergoing surgical resection at the Helsinki University Central Hospital (Helsinki, Finland). Patients included in the study were treatment naïve.

Patient samples were processed to fresh single-cell tumors. Tumors were diced into small fragments and placed in a 50 mL falcon tube containing RPMI 1640 (Sigma-Aldrich, St. Louis, MO, USA) supplemented with 1% L-glutamine, 1% Pen/strep (Gibco, Thermo Fisher Scientific, Waltham, MA, USA), collagenase type I (170 mg/L), collagenase type IV (170 mg/L), DNase I (25 mg/mL) (all enzymes from Worthington Biochemical, Lakewood, NJ, USA) for 2 h enzymatic digestion with rocking at +37 °C. After digestion, the cell suspension was filtered through a 100 μm filter and treated with Ammonium-Chloride-Potassium lysis buffer (Sigma-Aldrich, St. Louis, MO, USA) for the removal of undigested fragments and erythrocytes. The resulting single-cell suspension was used to establish ex vivo tumor cultures by plating fresh 0.35 × 10^6^ cells in a 96-well U-bottom plate followed by centrifugation at 300× *g* for 5 min. Wells (with supernatant intact) were then infected with 100 VPs/cell of either Ad5/3-E2F-D24 (from here referred to as the backbone virus) or Ad5/3-E2F-D24-hTNFa-IRES-hIL2 (TILT-123), anti-PD-1 (20 µg/mL), anti-PD-L1 (20 µg/mL) or medium (vehicle/neg. control). After incubation, the samples were analysed 1 to 7 days after incubation (marked in graphs) by flow cytometry or for viability. The use and collection of patient samples were approved by the ethics board of the University Hospital and was based on informed consent (ethic board approval number 120/03/02/16).

### 2.3. Immune Checkpoint Inhibitors

The following ICIs were used in the study: aRMP Bavencio (Cat# 8000471, Avelumab) -Merck Serono GmbH, aRMP Imfinzi (Cat# 8000488, Durvalumab)-Medimmune/AstraZeneca, aRMP Opdivo (Cat# 08000241, Nivolumab) Bristol-Myers Squibb GmbH & Co. KGaA were all purchased as aliquots (aRMPs) from Evidentic (Berlin, Germany). Tecentriq (Atezolizumab) and Keytruda (Pembrolizumab) were obtained from the Comprehensive Cancer Clinic, Helsinki, Finland. The mouse antibodies were purchased from BioXcell: BE0101-100MG InVivoMAb anti-mouse PD-L1 (B7-H1) (clone 10F.9G2) and BE0273-100MG InVivoMAb anti-mouse PD-1 (CD279) (clone RMP1.14).

### 2.4. Cell Viability

Cell viability was measured by incubating patient sample for 2 h with 20% of CellTiter 96 AQueous One Solution Proliferation Assay reagent (Promega, Madison, WI, USA). Absorbance was read at 490 nm using a Hidex Sense plate reader (Hidex, Turku, Finland). Data was normalized to the uninfected mock control group.

### 2.5. Chemokines and Cytokines Analysis

Supernatants from infected ex vivo tumor cultures were collected on days 3 and 7 and the presence of C-C motif ligand 2 (Ccl2), C-C motif ligand 5 (Ccl5), C-X-C motif chemokine 10 (Cxcl10), interleukin 2 (IL2), tumor necrosis factor alpha (TNFa), interferon gamma (IFNg), interferon 1 beta (IFN1b), interleukin 4 (IL4), interleukin 6 (IL6), interleukin 10 (IL10), and transforming growth factor beta-1 (TGF-b1) were determined using an Essential Immune Response LEGENDplex panel (Biolegend, San Diego, CA, USA) according to the manufacturer’s instructions.

Samples were measured in triplicates using the BD Accuri C6 flow cytometer (BD biosciences, San Jose, CA, USA) and analyzed using LEGENDplex Data Analysis Software Suite (Biolegend, San Diego, CA, USA). Data were normalized to the total protein content measured with Qubit4 Fluorometer (Invitrogen, Waltham, MA, USA).

### 2.6. Effect of TILT-123 Treatment on Tumor Microenvironment

For the analysis of immune cells from ex vivo tumor cultures, antibodies specific for human CD3 (clone SK7, Alexa Fluor 700, Biolegend), CD4 (clone RPA-T4, V500, BD Biosciences, NJ, USA), CD8 (clone RPA-T8, FITC, BD Biosciences, Franklin Lake, NJ, USA), CD69 (clone FN50, PE/Cyanine7, Biolegend), granzyme B (GrzmB) (clone GB11, BV421, BD Biosciences, NJ, USA), perforin (Perf) (clone B-D48, PerCP/Cyanine5.5, Biolegend, San Diego, CA, USA)), and CD107a (LAMP1) (clone H4A3, APC/Cyanine7, Biolegend, NJ, USA) were used. Intracellular staining was performed using BD Cytofix/Cytoperm Plus Kit (with BD GolgiPlug) (BD Biosciences). All samples were stained after Fc blocking using Human TruStain FcX Receptor Blocking Solution (Biolegend, San Diego, CA, USA). Samples were acquired in duplicates using FACS Aria II cell sorter (BD Biosciences, Franklin Lake, NJ, USA) and data analysis was performed using Flowjo software v10 (Flowjo LLC, BD Biosciences, Franklin Lake, NJ, USA).

### 2.7. Animal Model

For treatment efficacy studies, two subcutaneous tumors, 5 × 10^6^ cells per flank or intraperitoneal (i.p.) tumors (3 × 10^6^ cells) were implanted into C57BL/6NHsda mice (*N* = 6). Animals randomized into groups and treated i.p. by mock treatments (PBS, 100 µL), mouse anti-PD-1 (CD279) (NH BioXCell), or Equal amounts of Ad5-CMV-mTNFa and Ad5-CMVmIL2 (5 × 10^8^ VP of each virus per injection). The animals were treated nine times. The tumor size was followed by LAGO live imager by D-Luciferin i.p. injection (PerkinElmer, MA, USA, 3 mg/animal). Luciferin live imaging with LAGO (Spectral Instruments Imaging, Tucson, AZ, USA) was provided by The Bio Imaging Unit of University of Helsinki. The pictures were quantified by AURA (version 4.0.0.). The ROI values for each tumor was measured and percentual difference in growth compared to respective tumor on day 0 was calculated. The mean of % differences within one treatment group is shown. Comparison of TILT-123 to the “backbone vector” Ad5/3 has been carried out before [14] and, thus this group was not analysed in this study.

Experimental protocols and procedures were approved by the ethical committee of the Animal Experimental Board (ELLA) of the Regional State Administrative Agency of Southern Finland, license number ESAVI/28404/2019.

### 2.8. Statistical Analysis

Prism 8 (GraphPad Software, San Diego, CA, USA) was used for statistical analysis. *p* values < 0.05 were considered significant. Viability tests and T cell maturation status tests were performed in triplicates, while cytokines were measured twice in duplicates. Animal experiments were performed with *N* = 6.

## 3. Results

### 3.1. Anti-PD-1 and Oncolytic Adenovirus Treatment Kills Patient Tumor Cells Efficiently

**Figure 1:**
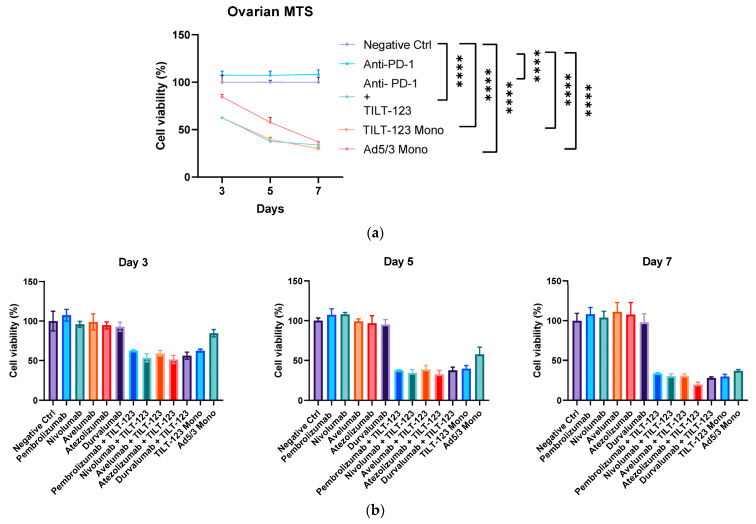
Ovarian cancer cell viability after virus and/or immuno-checkpoint inhibitor treatments. (**a**) HUSOV4 patient sample cancer cells were treated with; media (negative control), Pembrolizumamb 20 mg/mL, TILT-123 virus (100 VP/cell), or with a combination of pembrolizumab plus TILT-123 virus. Controls were left untreated. (**b**) TILT-123 virus cytotoxicity in ovarian cancer cells when administrated as a monotherapy or in combination with most commonly used human PD-1 and PD-L1 antibodies. All ICI antibodies were used as 20 mg/mL. MTS assay was performed as mentioned above, with same concentrations but several different ICI. Statistics; One way ANOVA (Tukey’s multiple comparison) was used for statistics on day 7 (**** *p* ≤ 0.0001). Data has been gathered as triplicates and is presented as mean + SEM.

In order to see the efficacy of TILT-123 treatment alone or in combination with ICIs, ovarian cancer patient samples were infected with TILT-123 and/or treated with anti-PD-1/PD-L1 (Figure 1A,B). Anti-PD-1 treatment alone did not reduce the viability of the cancer cells. However, both TILT-123 monotherapy and combinatory treatments showed efficient killing; by day 5 the viability of double treated sample dropped to under 40% and by day 7 only 33% of cancer cells were viable in the combination group (Figure 1, mock vs. TILT-123+ anti-PD-1 *p* < 0.001). Logically, in line with studies by Santos et al., some stromal cells are not infected and killed by the cancer cell specific oncolytic adenovirus treatment and thus a 100% killing cannot be achieved in this test setup [22].

Cell viability was not affected by any ICI treatment alone, while the combination of ICI and TILT-123 caused an efficient cell killing; less than 40% of cells were viable on day 7, with no difference between the ICI + virus groups. The addition of transgenes to the adenovirus vector caused an increase in cell death by 10–20%, depending on the measured time point. This is plausibly due to TNFa as it is a known necrosis factor. The noted limited effect of ICI might be due to systematic reasons, with no lymph nodes with APC to train the immune compartment. Additionally, as it is an in vitro setup, normally (in patents) viable cells start to die, thus the effects of the immune system cannot be seen as potently in this setup.

### 3.2. Oncolytic Adenovirus and ICI Therapy Induced Change in the Cytokine Environment

**Figure 2:**
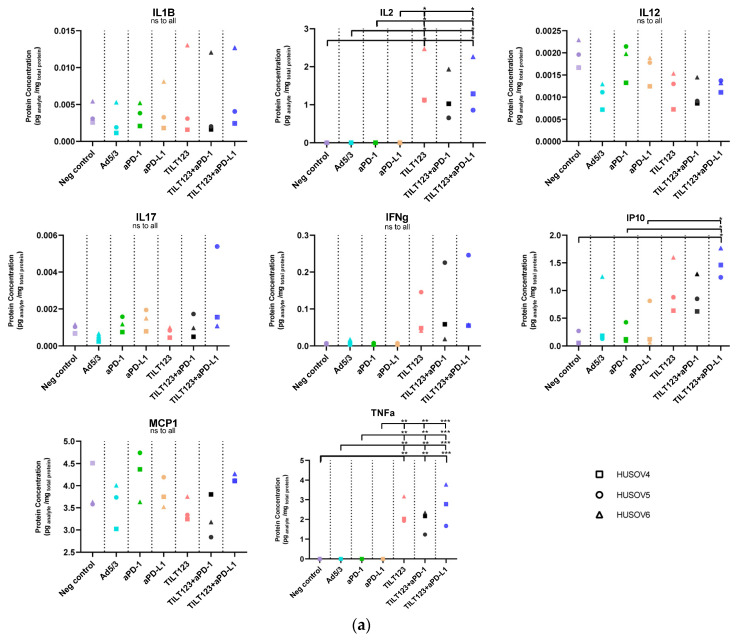

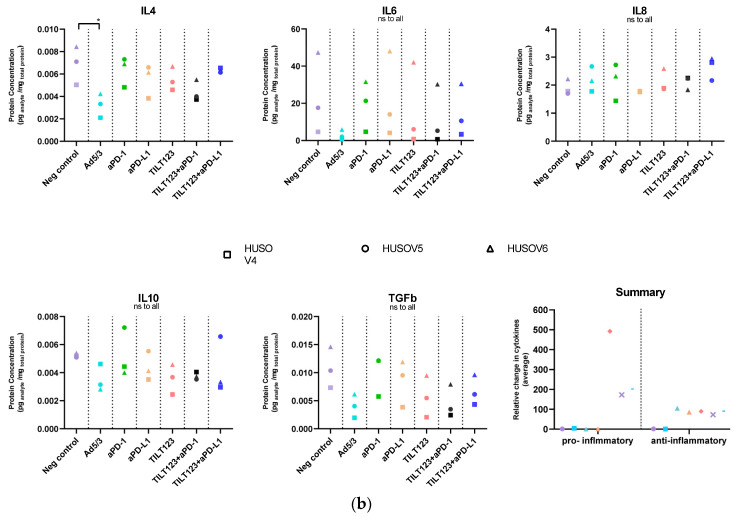
Cytokine concentration changes in response to virotherapy and or ICI treatments. Three patient tumor samples (HUSOV4- HUSOV6) were treated with backbone virus, TILT-123, anti-PD-1, anti PDL-1, or a combination of TILT-123 and checkpoint inhibitor. (Viruses 100 VP/mL, ICI 20 mg/mL). Fresh media functioned as a control condition. The cytokine concentrations were measured from the supernatants on day 7 by flow cytometric assay. (**a**) Pro-inflammatory cytokines (**b**) Anti-inflammatory cytokines and summary of A &B combined. Statistics; One way ANOVA (Tukey’s multiple comparison) was used for statistics, with * *p* ≤ 0.05, ** *p* ≤ 0.01, *** *p* ≤ 0.001, data have been gathered from triplicates.

Ovarian cancer patient samples were analysed for cytokine concentration changes after adenovirus treatments, with or without anti-PD1/anti-PD-L1 (pembrolizumab and atezolizumab) (Figure 2).

In 1/3 samples, the IL1b concentration rose markedly on both day 3 and 7 in TILT-123 and combination treated samples; however, this could not be reproduced in the other samples and was not statistically significant.

ICIs and unarmed backbone viruses did not cause an IFNg release. The combination groups and TILT-123 caused a slight trend of higher release of IFNg in 3/3 samples on both day 3 and 7 compared with the control group; however, this was not statistically significant.

Logically, since TILT-123 encodes for IL2 and TNFa, the TILT-123 treated groups showed high concentrations of IL2 on both days 3 and 7 which was kept also in the double treated groups (day seven IL2: mock vs. TILT-123 *p* = 0.0156, or anti-PD-1 *p* = 0.0848, vs. TILT-123+ anti-PD-L1 *p* = 0.0251, day 7 TNFa: mock vs. TILT-123 *p* = 0.011 or TILT-123 + anti-PD-1 *p* = 0.0076, or vs. TILT-123 + anti-PD-L1 *p* = 0.0003).

IP10 was statistically significantly higher in samples treated with TILT-123 in combination with anti-PD-L1 compared to mock treatment (*p* = 0.0104).

No statistically significant changes in MCP1, IL12 and IL17 could be measured.

Interestingly, out of the anti-inflammatory cytokines (Figure 2B) TILT-123 treatment caused a slight trend of lowered IL10 secretion in all treated patient samples on day 7, and the combination of TILT-123 and anti-PD-1 (not anti- PD-L1) enhanced it further. Backbone treatment caused a statistically significant difference in release of IL4, while other treatments did not affect the secretion of IL4.

In summary, we can see that the combinatorial treatment enhances the concentration of pro-inflammatory cytokines, giving a rationale to TILT-123 + ICI therapy.

### 3.3. TILT-123 and Combinatorial Treatment of Ex Vivo Patient Ovarian Tumor Samples Changes the Activation Status and Number of Patient T Cells

**Figure 3:**
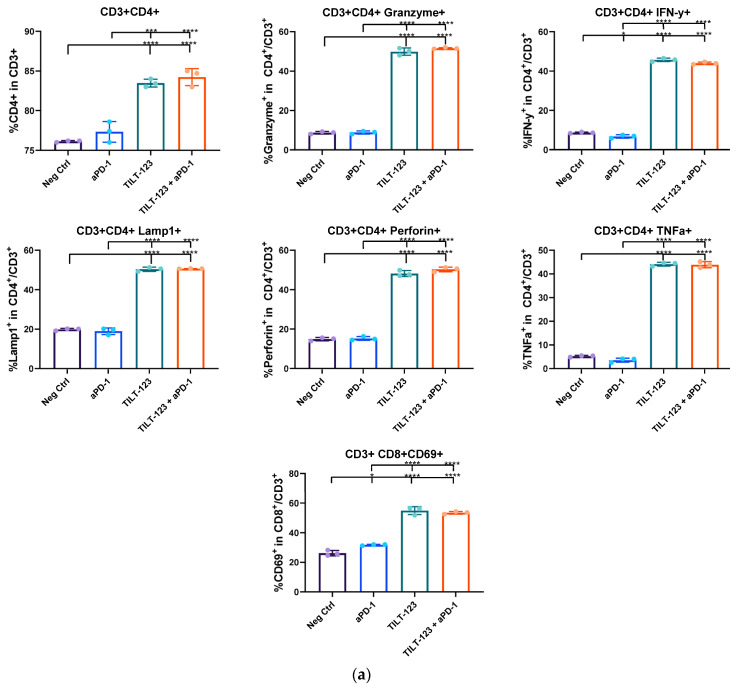

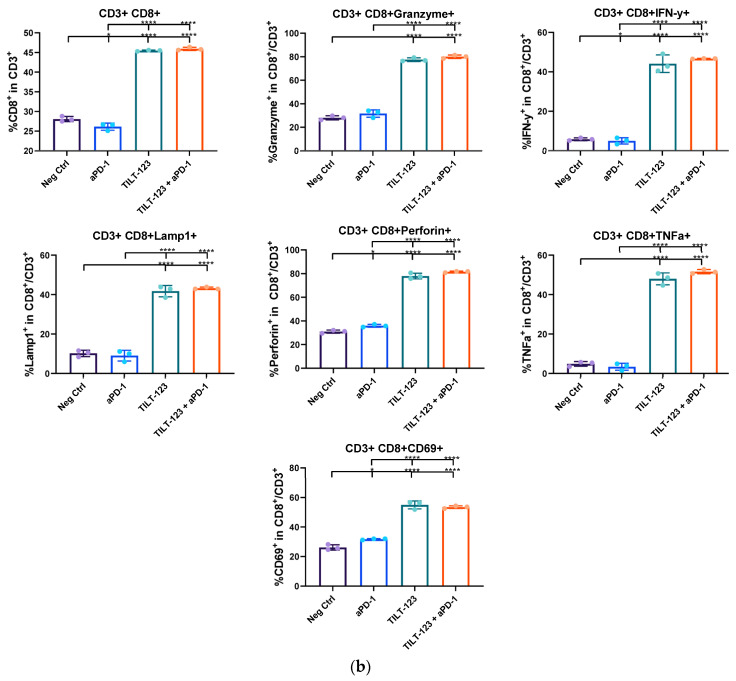
T cell responses to in vitro; viro- and checkpoint inhibitor therapy. T cells isolated from human patient samples were incubated in the stated settings. The response to the treatment was measured by flow cytometry of triplicates. Data for (**a**) CD4 positive cells and for (**b**) CD8 positive cells.Statistics; One way ANOVA (Tukey’s multiple comparison) was used for statistics, with * *p* ≤ 0.05, *** *p* ≤ 0.001, **** *p* ≤ 0.0001 Combinatorial treatments halt tumor growth or cure animals bearing ovarian cancer tumors.

Processed single cell samples of patient tumors were treated with TILT-123 and/or ICI or control treatments, to gain mechanistic insight into the previously noted cancer cell killing. The samples were gated to contain only lymphoid cells and analysed for their activation markers (Figure 3, Gating strategy shown in Supplementary data B).

Samples treated with backbone virus, TILT-123 only, or with both TILT-123 virus and ICI, showed a significantly higher % of CD3+ CD4+ and CD3+ CD8+ T cells compared to mock (*p* ≤ 0.0001), while treatment with only ICI did not cause a significant effect on day 7.

TILT-123 with or without anti-PD-1 caused similar reactions in both CD4+ and CD8+ cells. In all virotherapy or combination treated groups the expression of CD69+, perforin, granzyme b, lamp-1 and IFNg were statistically significantly higher compared to mock (*p* ≤ 0.0001). Overall, the results were similar, but not as strong on day 3 (Supplementary data A). Thus, it seems that the virus in itself causes the growth in the immune population.

Interestingly, when a significant rise in the percentage of double positive lymphocytes (CD4+ and CD8+ out of CD3+ cells) in patient samples treated with virotherapy and/or ICI was analysed, a significant a rise in the percentage of double positive cells could be seen compared to negative control (see Supplementary data C).

**Figure 4:**
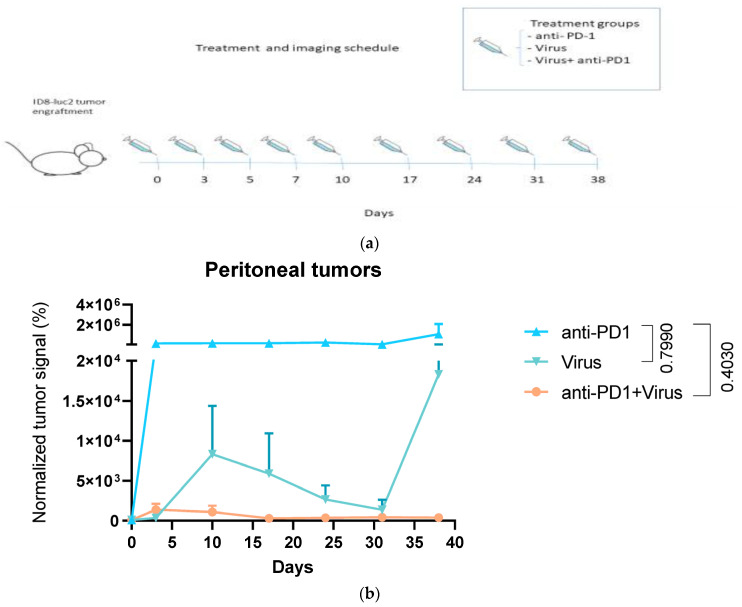
Tumor volume changes in response to treatments. (**a**) The normalized tumor volume was followed according to the schedule described in the picture. (**b**) The animals were imaged by LAGO after an 3 mg luciferin i.p. injection and the normalized tumor signal was calculated by comparison to the tumor signal of the animal on day 0 of treatments. Kruskal-Wallis test was performed on the last day of measurements, *N* = 6.

In order to verify the effect of TILT-123 and anti-PD-1 treatment on tumor growth in vivo, mice were implanted subcutaneously (Supplementary data D) and intraperitoneally (Figure 4) with ID8-luc2 tumors. The treatments were administered every other or third day for 5 treatments (10 days), and continued once a week until day 38. Tumor growth was measured by luciferase emission. In order to see the effects of TILT-123’s cytokines in the murine model, adenoviruses coding for murine cytokines were used. The measurements showed a trend that the subcutaneous tumor burden of virus and combinatorial treatment animals (anti-PD-1 and TILT-123) was reduced compared to anti-PD-1 only. The positive tumor reducing effect was even more evident when intraperitoneal tumor burdens were measured. The normalized burden of anti-PD-1 treated mice was 15 000-fold higher compared with combination treatments (not statistically significant, Kruskal- Wallis anti-PD-1 vs. anti-PD-1 +TILT-123 *p* = 0,403). Notably, 1/6 TILT-123 treated and in 3/6 combination treated animals (peritoneal results), showed no measurable luciferase signals on day 38 of treatment, which could indicate that the animals might have been cured or that the tumor volume had decreased beneath detectable size.

## 4. Discussion

Broadly speaking, tumors can be divided into two different categories according to their immune infiltration and inflammation status: immunologically hot and cold tumors. This classical division has later been refined into a theory of immune infiltrated (hot), immune excluded (infiltration in the periphery of the tumor) and immune deserted (cold) tumor models. The T cells that are already present in hot tumors can be activated with the help of ICIs and, thus, they should theoretically benefit the most from ICI treatment. However, the efficacy of immunotherapies vary even in these tumor types. This can be seen, for example, in the modest results of a phase III clinical trial, where pembrolizumab and ipilimumab regimes were compared. In this study, 834 patients with advanced melanoma were enrolled and randomly assigned to receive pembrolizumab either every 2 weeks (*N* = 279) or every 3 weeks (*N* = 277). A third group of patients received ipilimumab (*N* = 278). In a close to five years’ follow up, the median overall survival was 32.7 months in the combined pembrolizumab groups and 15.9 months in the ipilimumab group. Median progression-free survival was 8.4 months in the combined pembrolizumab groups while it was much shorter, 3.4 months, in the ipilimumab group [23].

On the other hand, cold tumors, with few if any T cells, do not usually benefit from checkpoint inhibition, simply because there are only few if any T cells for ICIs to apply their effect. Some results indicate that prostate cancer and pancreatic cancer are typically cold tumors, limiting the benefit gained from immunotherapeutic regimes [11], while ovarian cancer is placed in the analysis somewhere in the middle, as per having a median value of somatic mutations (Alexandrov et al. 2013). Thus, OVs have been employed to attract immune cells to the tumor by modulating the tumor micro-environment to a more ICI permissive state, in other words, for the development of a hot tumor [14]. For example, it has been shown that OVs cause neoantigen release, danger and pathogen associated molecular pattern signaling in addition to their cancer cell killing lytic effects [24,25]. This causes the tumor microenvironment to change and attract lymphocytes while the virus itself also de-bulks some tumor mass. This reaction is then hoped to be further enhanced by the addition of ICIs in this study.

To capture the complexity present in the tumor microenvironments we used human patient samples. While interpreting these findings, it should be noted that results obtained in disaggregated cells cannot automatically be assumed also to apply to whole tumors.

Here, we noted that TILT-123, with or without ICIs, killed ovarian cancer patient tumor-derived cells efficiently. One can speculate that the remaining cells could be non-cancerous cells, such as fibroblasts or immune cells. For example, in a study where cancer cell lines were developed from patient samples, authors reasoned that stroma outgrowth was one of the major reasons why they could not always establish cell lines [26]. Additionally, further explanation as to why no synergy was seen could be related to lack of or limited amount of active cytotoxic cells in the sample on which ICI function The main difference affecting the results between TILT-123 and the Ad5/3 are probably due to the cytokine encoding genes (hTNFa and hIL2) that have been added to the E3 region of the oncolytic adenovirus in TILT-123. As TNFa is known as a potent tumor necrosis factor, the local, high concentrations of TNFa in the in vitro study has likely caused some cell killing [27].

IL2 is mainly known for its effects on T cells (differentiation and growth); however, many nonlymphoid cell types also express the IL-2R. Unfortunately, less information on its effect on these cells is available. What is known, however, is that it has a strong impact on gastrointestinal epithelial cells, endothelial cells, and fibroblasts [28]. In studies where IL2 production or its receptor were blocked, effects were seen on mouse smooth muscle, and it also caused vascular leakage through gap formation in endothelial cell layers (loss of cell-to-cell contact) and cell damage [29]. Therefore, it is likely that the IL2 produced by TILT-123 causes some additional loss of cell viability. These differences will probably be highlighted in test setups with immune cells, which additionally react to the produced cytokines. Thus, this test setup showed proof of concept and rationale to continue with the further tests. The positive cancer cell killing result encouraged us to take a further look into the changes in the tumor microenvironment, in order to understand the reaction better. The concentration of IP10 was significantly higher in the group treated with both anti-PD-L1 and TILT-123. IP10 is a chemokine, also known as CXCL10, which attracts several cell types, such as lymphocytes and natural killer cells. Studies have shown that high CXCL10 concentrations in advanced serous ovarian cancer patients correlated with better survival through attraction and infiltration of T cells [30], thus encouraging the use of combinatorial treatments. We measured high concentrations of TNFa, IL-2 and IFNg in samples treated with TILT-123 and/or anti-PD-1. These three cytokines all contribute to positive T cell reactions, such as T cell activation and proliferation. These cytokine measurements indicate that the changes made with the treatments could result in cold tumors becoming hot, which could eventually favor patient outcomes in the context of ICI therapy.

After grouping the measured cytokines into pro- and anti-inflammatory cytokines and seeing the change in their relative expression, it is evident that TILT-123 alone, and the combination treatment with anti-PD-1, triggers the release of pro-inflammatory cytokines, which the ICI treatments alone or the backbone virus were not able to do. Simultaneously, as often noted, every action in immunology triggers a counter response. Thus, all of the treatments, which are historically thought of as pro-inflammatory, also triggered a slight relative rise in the release of anti-inflammatory cytokines.

Activated T cells secrete IFNg, granzymes and have high lamp-1 and CD69 expression. IFNg directly acts as a cytotoxic CD8 T cell differentiation signal, and it is essential for the induction of cytotoxic T cell precursor proliferation. Additionally, IFNg regulates cell surface MHC class II on APCs, thus promoting peptide-specific activation of CD4 T cells. This indicates that, if TILT-123 and ICI treatments are given together, T cells will be primed and activated for killing of cancer cells [31]. As the tumor microenvironment changes are linked to patient outcome, it would be interesting to conduct further studies for more information on the subject.

When studied in vivo, we saw a positive tumor debulking effect when double treatment was given to tumor bearing mice, thus further supporting the use of combination treatment in clinical trials. To compensate for species incompatibilities, we utilized multiple injections, and thus an optimized treatment frequency should be evaluated in future studies, and could further improve results. Interestingly, the luciferase signal for 1/6 TILT-123 treated and in 3/6 combination treated animals decreased below measurable values, indicating that the animals were possibly cured. In this study it was unfortunately not possible to collect tumor samples for, e.g., immunological analyses. However, we consider such analyses valuable and therefore they could be performed in a follow-up study. The comparison of treatments to untreated animals has been performed before, and was thus not conducted in this study [14,20].

All in all, the data in this study suggests that TILT-123 causes a change in the tumor microenvironment. It acts as a two-pronged attack, debulking the tumor by immune stimulation and direct lysis. Of note, recent data suggests that this approach acts also on hard-to-reach distant tumors and metastases, through the abscopal effect [14,32,33]. The lysis of infected cells releases danger and pathogen associated molecules together with pro-inflammatory cytokines, attracting T cells. [20,25]. Then, when T cells infiltrate the tumor, they are more active to cytotoxic actions as the virus induced milieu change triggers them into action. This immune activation and infiltration has also been seen in other adenoviral therapies [34], e.g., in n in vivo mouse model, the adenovirus vector Delta-24-RGDOX. In the study made by Jiang et al., potent anti-glioma activity was reported in immunocompetent C57BL/mice. This was not seen in immunodeficient athymic mice, suggesting specific immune memory against the tumor [35].

When the tumor is simultaneously treated by checkpoint inhibitors, the treatment might function as a boosting agent to upkeep this T cell reaction resulting in a synergistic outcome. As the goal of ICI is to activate the immune system to fight cancer, logically, the administration of ICI has been shown to cause some autoimmune reactions, manifesting usually as mild adverse events [36]. However, this phenomenon can be thought as potentially beneficial in our treatment setup. When the virus lyses cells, TAA and other non-cancer specific antigens are released and T cells react on them, thus the concomitant administration can be thought to boost the efficacy of ICI in this way too.

Thus, this treatment regime could help patients that have hard to treat resistant tumors and could be a solution to the lack of effective therapies available to ovarian cancer patients with platinum refractory disease. Based on the preclinical work reported here, and other pertinent data [14,20,22], a clinical trial is under way, testing the combination of anti-PD-1 and TILT-123 in platinum refractory ovarian cancer patients.

## 5. Conclusions

In conclusion, in this preclinical study, we found that TILT-123 treatment in combination with ICI is an attractive new treatment modality for treatment of ovarian cancer in clinical trials (NCT05271318).

## Figures and Tables

**Table 1:** Patient sample material.

Sample Name	Age	Initial Location	Metastases	Location of Sampling
HUSOV4	79	High grade serous carcinoma of the fallopian tube St IVB	Peritoneum	Greater omentum
HUSOV5	76	High grade serous carcinoma of the fallopian tube St IVB	Peritoneum	Greater omentum
HUSOV6	35	Low grade serous carcinoma of ovary St IVB	Peritoneum	Greater omentum

## Data Availability

Not applicable.

## Supplementary Materials

The following supporting information can be downloaded at: https://www.mdpi.com/article/10.3390/diseases10030052/s1.
