# Supplementary material for: Effective Combination Immunotherapy with Oncolytic Adenovirus and Anti-PD-1 for Treatment of Human and Murine Ovarian Cancers
Source: Diseases. 2022 Aug 8;10(3):52. doi: 10.3390/diseases10030052 (PMC9396998; doi:10.3390/diseases10030052)
Supplement: Supplementary file 1 [file diseases-10-00052-s001.zip › diseases-1756373-supplementary.pdf]

Supplementary Data A

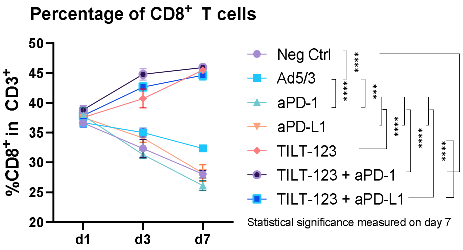

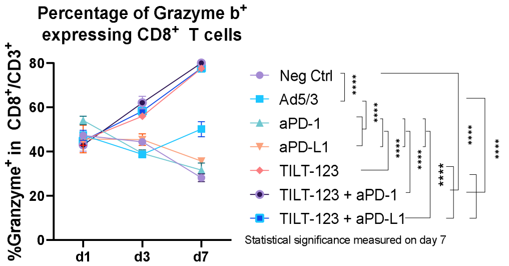

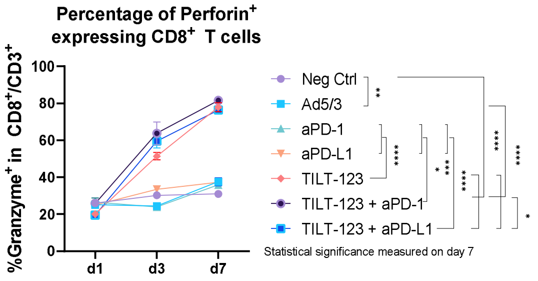

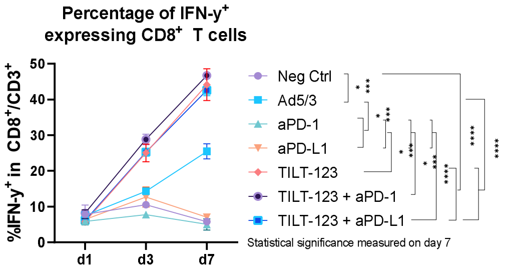

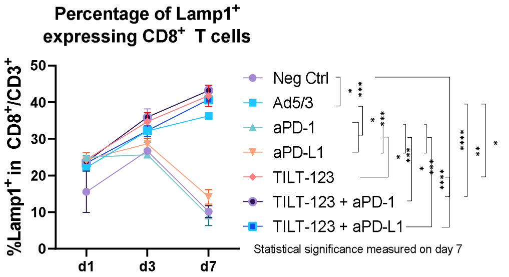

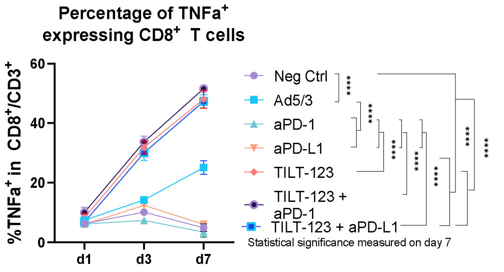

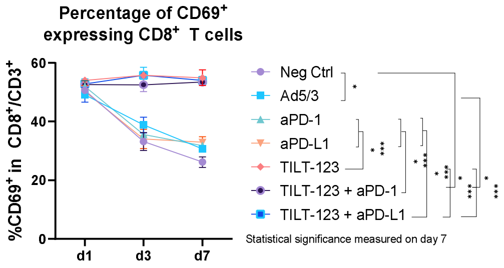

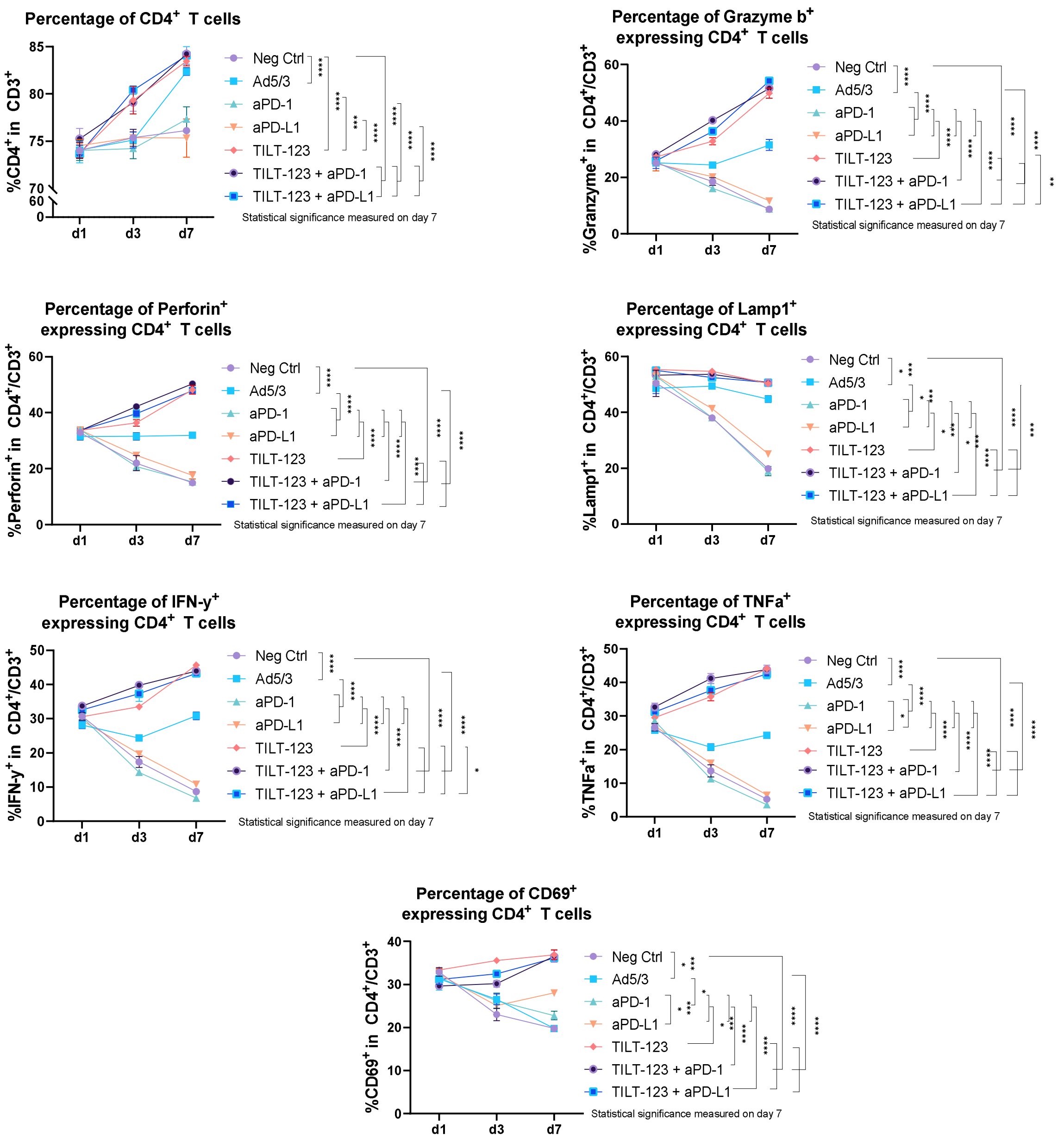

Supplementary data A. T cell responses to vitro- and chekpoint inhibitor therapy. T cells isolated from human patient samples were incubated in above stated settings. The response to the treatment was measured by flow cytometry. Statistics; One way ANOVA (Tukey's multiple comparison) was used for statistics, with * $p \leq 0.05$,** $p \leq 0.01$,*** $p \leq 0.001$,**** $p \leq 0.0001$.

Supplementary data B

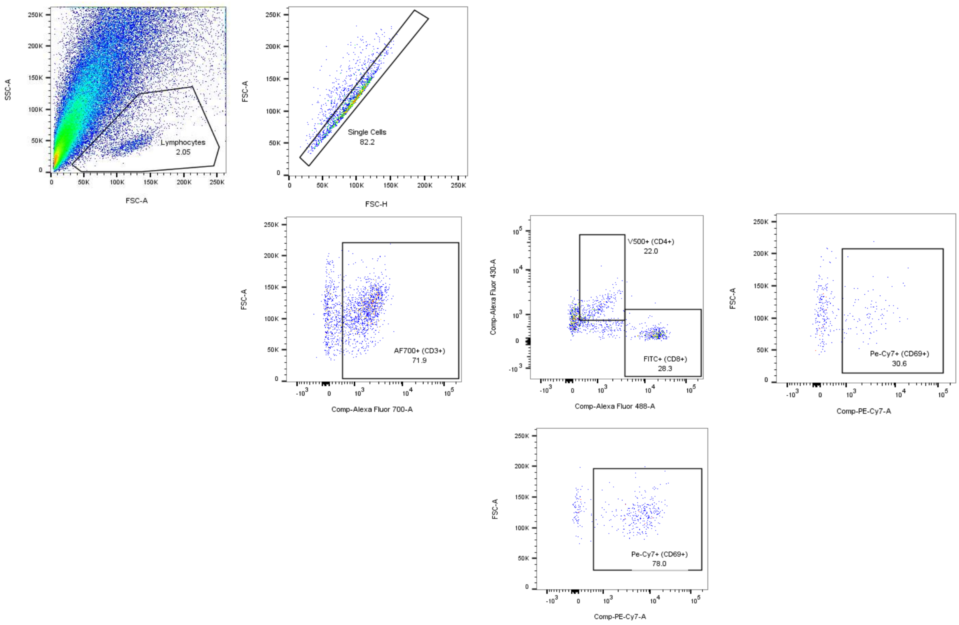

Supplementary data B. Gating strategy for human patient sample T cell composition and activation in Figure 3 and data A.

Supplementary data C

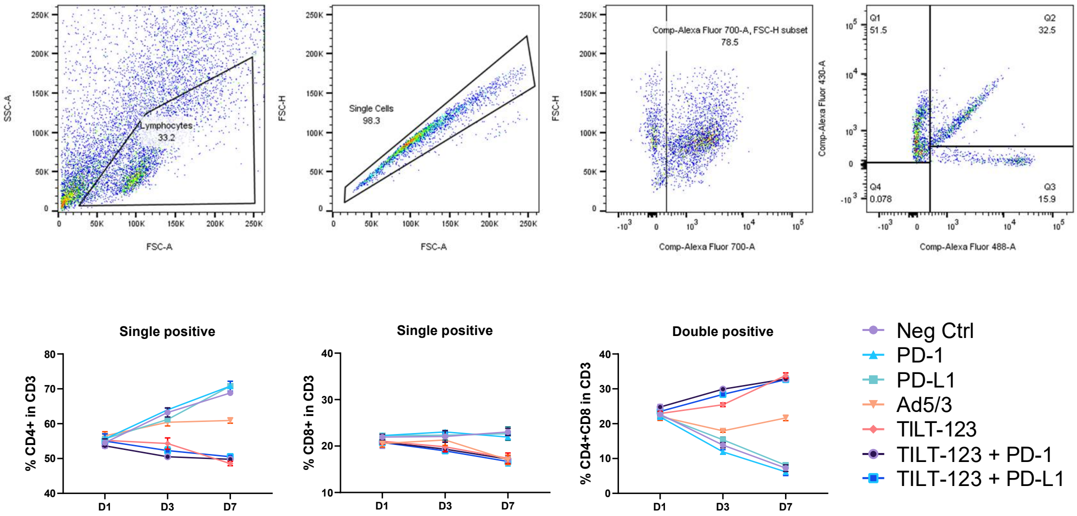

	Single CD4 %		Single CD8%		Double CD4+ & CD8+ %	
Tukey's multiple comparisons test	Adjusted P Val	Summary	Adjusted P Val	Summary	Adjusted P Val	Summary
Neg Ctrl vs. PD-L1	0,1994	ns	0,999	ns	0,7993	ns
Neg Ctrl vs. PD-1	0,1994	ns	0,8016	ns	0,5909	ns
Neg Ctrl vs. Ad5/3	<0,0001	****	<0,0001	****	<0,0001	****
Neg Ctrl vs. TILT-123	<0,0001	****	<0,0001	****	<0,0001	****
Neg Ctrl vs. TILT-123 + PD-1	<0,0001	****	<0,0001	****	<0,0001	****
Neg Ctrl vs. TILT-123 + PD-L1	<0,0001	****	<0,0001	****	<0,0001	****
PD-L1 vs. PD-1	>0,9999	ns	0,9625	ns	0,0797	ns
PD-L1 vs. Ad5/3	<0,0001	****	<0,0001	****	<0,0001	****
PD-L1 vs. TILT-123	<0,0001	****	0,0002	***	<0,0001	****
PD-L1 vs. TILT-123 + PD-1	<0,0001	****	0,0001	***	<0,0001	****
PD-L1 vs. TILT-123 + PD-L1	<0,0001	****	<0,0001	****	<0,0001	****
PD-1 vs. Ad5/3	<0,0001	****	0,0004	***	<0,0001	****
PD-1 vs. TILT-123	<0,0001	****	0,0008	***	<0,0001	****
PD-1 vs. TILT-123 + PD-1	<0,0001	****	0,0005	***	<0,0001	****
PD-1 vs. TILT-123 + PD-L1	<0,0001	****	0,0002	***	<0,0001	****
Ad5/3 vs. TILT-123	<0,0001	****	0,9994	ns	<0,0001	****
Ad5/3 vs. TILT-123 + PD-1	<0,0001	****	>0,9999	ns	<0,0001	****
Ad5/3 vs. TILT-123 + PD-L1	<0,0001	****	0,9994	ns	<0,0001	****
TILT-123 vs. TILT-123 + PD-1	0,6787	ns	>0,9999	ns	0,6582	ns
TILT-123 vs. TILT-123 + PD-L1	0,186	ns	0,9762	ns	0,4688	ns
TILT-123 + PD-1 vs. TILT-123 + PD-L1	0,9385	ns	0,9945	ns	0,9999	ns

Supplementary data C. T cell responses and gating strategy to vitro- and chekpoint inhibitor therapy. T cells isolated from human patient samples were incubated in above stated settings. The response to the treatment was measured by flow cytometry. Statistics; One way ANOVA (Tukey's multiple comparison) was used for statistics, with ,*** $p \leq 0.001$, **** $p \leq 0.0001$.

Supplementary data D

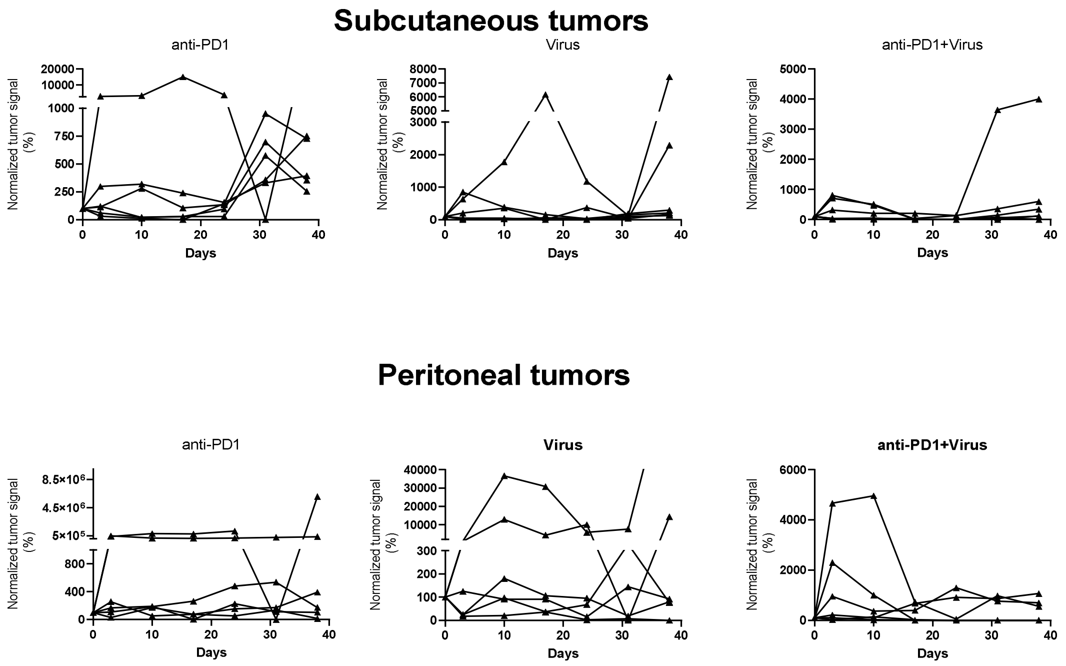

Supplementary data D. Individual tumor growth curves. Animals treated as stated in Figure 4 and Materias and methods. Individual tumors measured with LAGO bioimager.
